# Comparative transcriptome analysis of *Sclerotinia sclerotiorum* revealed its response mechanisms to the biological control agent, *Bacillus amyloliquefaciens*

**DOI:** 10.1038/s41598-020-69434-9

**Published:** 2020-07-28

**Authors:** Xiaoxiang Yang, Lei Zhang, Yunjia Xiang, Lei Du, Xiaoqin Huang, Yong Liu

**Affiliations:** 1 Institute of Plant Protection, Sichuan Academy of Agricultural Sciences, Chengdu, 610066 Sichuan People’s Republic of China; 2 Key Laboratory of Integrated Pest Management on Crops in Southwest, Ministry of Agriculture and Rural Affairs, Chengdu, 610066 Sichuan People’s Republic of China; 3 Present Address: Sichuan Academy of Agricultural Sciences, 20 # Jingjusi Rd, Chengdu, 610066 Sichuan People’s Republic of China

**Keywords:** Transcriptomics, Antimicrobials, Pathogens

## Abstract

Biological control mechanisms of plant diseases have been intensively studied. However, how plant pathogens respond to and resist or alleviate biocontrol agents remains largely unknown. In this study, a comparative transcriptome analysis was performed to elucidate how the pathogen of sclerotinia stem rot, *Sclerotinia sclerotiorum*, responds and resists to the biocontrol agent, *Bacillus amyloliquefaciens*. Results revealed that a total of 2,373 genes were differentially expressed in *S. sclerotiorum* samples treated with *B. amyloliquefaciens* fermentation broth (TS) when compared to control samples (CS). Among these genes, 2,017 were upregulated and 356 were downregulated. Further analyses indicated that various genes related to fungal cell wall and cell membrane synthesis, antioxidants, and the autophagy pathway were significantly upregulated, including glucan synthesis, ergosterol biosynthesis pathway, fatty acid synthase, heme-binding peroxidase related to oxidative stress, glutathione S-transferase, ABC transporter, and autophagy-related genes. These results suggest that *S. sclerotiorum* recruits numerous genes to respond to or resist the biocontrol of *B. amyloliquefaciens*. Thus, this study serves as a valuable resource regarding the mechanisms of fungal pathogen resistance to biocontrol agents.

## Introduction

Biological control is an effective way by using beneficial microorganisms or microbial metabolites to control plant diseases. In the biological control system, antagonism is one relationship exists between biocontrol agents and plant pathogens. Previous studies mainly focused on the biosynthesis of bioactive substances and antimicrobial mechanisms, while the stress responses of plant pathogens under the pressure of biocontrol agents have often been ignored. Under the stress of fungicides, plant pathogens alleviate the toxic reaction of fungicide by various pathways to develop resistance^1,2^. Likewise, plant pathogens elicit resistance under the continuous stress of biocontrol agents. However, most of the underlying mechanisms of resistance remain unknown.

As an excellent biocontrol agent, *Bacillus amyloliquefaciens* exhibits a broad spectrum of antifungal activities with the ability to produce different types of cyclic lipopeptides. Cyclic lipopeptides produced by *B. amyloliquefaciens* are catalyzed by non-ribosomal peptide synthase^3^, which inhibit the growth of various plant pathogenic bacteria and fungi ^4–7^. Cyclic lipopeptides include lipopeptide surfactin, iturins, and fengycins^8^. Lipopeptide surfactin exhibits strong surface and biological activities, emulsification and foaming properties. Due to the amphipathic features of lipopeptide surfactin, it anchors into the lipid layer and damages the integrity of biofilm under a certain dosage^9^. Iturin A and C; bacillomycin D, F, L, and LC; and mycosubtilin are seven main variants within the iturin family, which exhibit strong antifungal activities by increasing the permeability of the membrane^10^. Fengycin is a mixture of isoforms that is divided into two groups, A and B, based on the amino acid sequence of the peptide moiety, and exhibits strong antifungal activities, which interacts with the lipid layer and changes the structure and permeability of membrane^11^.

*Sclerotinia sclerotiorum* is a worldwide plant pathogen that causes sclerotinia disease on many economically important crops, including oilseed rape, lettuce, carrots, vegetable brassicas, peas, and beans, resulting in huge economic losses every year^12,13^. According to an investigation conducted on the national rape industry technology system, the annual loss of rapeseed caused by *S. sclerotiorum* in the Yangtze River basin in China exceeded 2 billion yuan. Application of resistant varieties is the most safe, environmentally friendly, and efficient way to control plant disease. However, no sclerotinia disease-resistant germplasm resources are readily available or in production. Currently, chemical control is the main control measure used for oilseed rape sclerotinia disease. However, long-term use of chemical pesticides leads to the generation of resistant strains and environmental pollution. Therefore, the development of environmentally friendly biological control measures is ideal for managing sclerotinia stem rot on oilseed rape, and is also in line with the national policy of "reducing fertilizer and reducing pesticide" and the national strategic demand of building ecological civilization in China.

Previously, *B. amyloliquefaciens* strain Bam22 was isolated from oilseed rape rhizosphere in Sichuan province, China, which exhibited the strong ability to inhibit the growth of *S. sclerotiorum*. On potato dextrose agar (PDA) plate containing the fermentation broth of Bam22, the *S. sclerotiorum* mycelial growth was inhibited and turned into "balloons". The morphological change of *S. sclerotiorum* may be a result of cyclic lipopeptides produced by *B. amyloliquefaciens.* However, how *S. sclerotiorum* responds to the toxicity of *Bacillus* (i.e., cyclic lipopeptides) remains unclear.

In this study, *B. amyloliquefaciens* and *S. sclerotiorum* were used to explore the response of plant pathogens against biocontrol agents. Transcriptome sequencing technology was used to compare and analyze the gene expression of *S. sclerotiorum* between wild-type strain 1980 and strain 1980 treated with the fermentation broth of *B. amyloliquefaciens*. Thus, the goal of this study was to decipher the response mechanisms of *S. sclerotiorum* to *B. amyloliquefaciens* stress at the transcriptome level.

## Results

### Bam22 inhibits the growth of *S. sclerotiorum*

**Figure 1:**
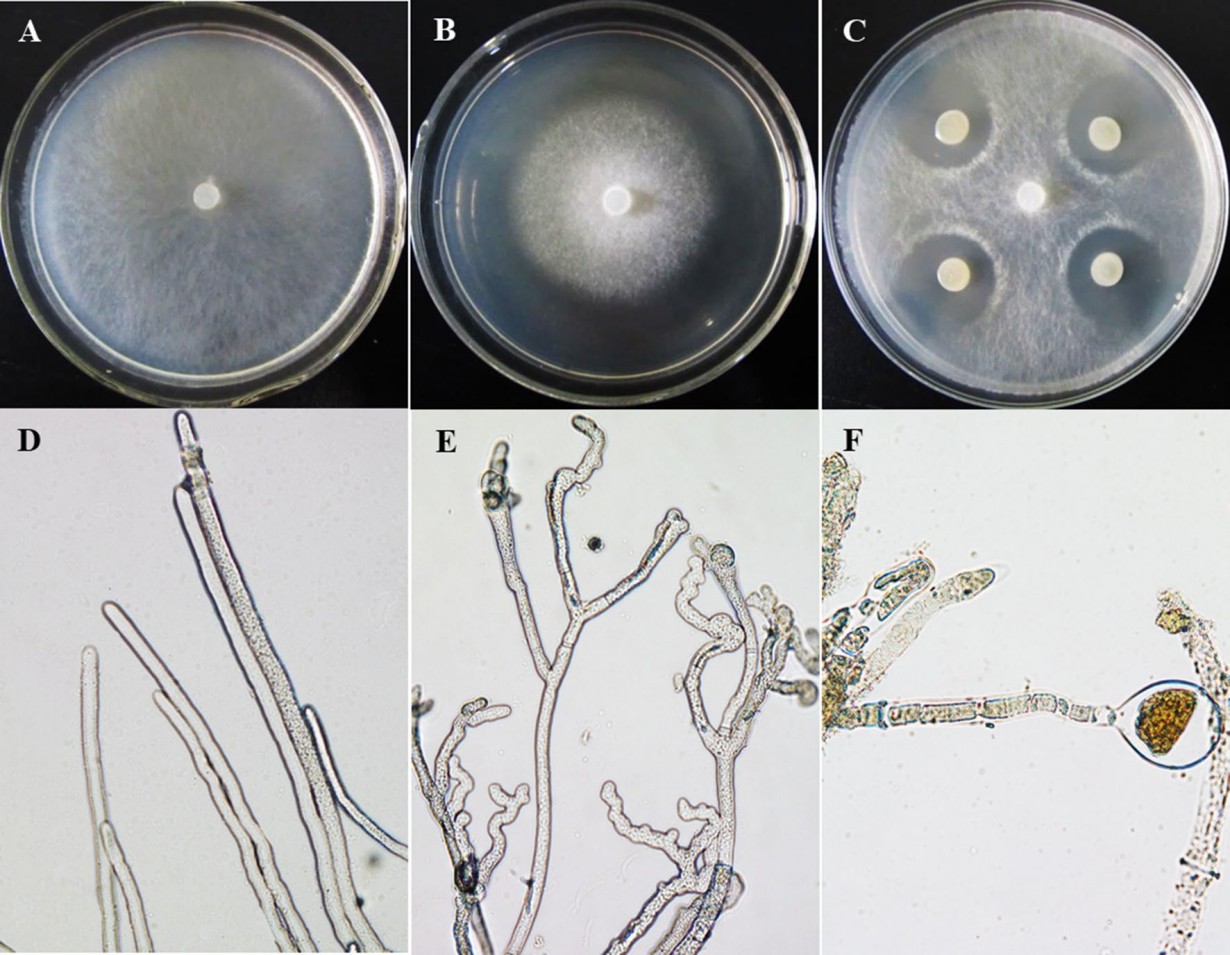
Bam22 inhibited the growth of *S. sclerotiorum*. (**A**) Colony morphology of *S. sclerotiorum* cultured on a PDA plate, (**B**) on a PDA plate that contained the fermentation broth of Bam22, and (**C**) dual cultured with *B. amyloliquefaciens* strain Bam22. (**D**) Hyphae morphology of *S. sclerotiorum* cultured on a PDA plate. (**E**,**F**) Hyphae morphology of *S. sclerotiorum* cultured on a PDA plate containing the fermentation broth of Bam22.

After dual culturing at 20 °C for 2 d, *B. amyloliquefaciens* strain Bam22 significantly inhibited the growth of *S. sclerotiorum* strain 1980 with a clear inhibition zone (Fig. 1). On PDA plate containing the fermentation broth of Bam22, the mycelial growth of *S. sclerotiorum* was inhibited and the top of the hyphae were abnormal, swollen, or turned into "balloons" (Fig. 1). These results indicated that strain Bam22 was able to inhibit the growth of *S. sclerotiorum* and change its hyphae morphology.

### Gene expression characteristics of *S. sclerotiorum* under *B. amyloliquefaciens* stress

**Figure 2:**
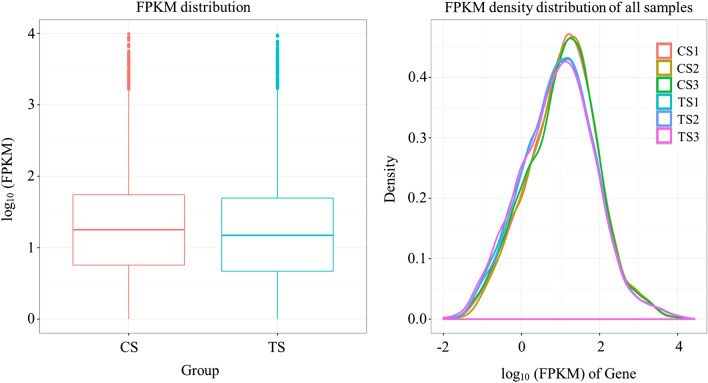
Comparison of the FPKM distribution and FPKM density distribution of all samples.

In order to decipher the changes of *S. sclerotiorum* gene expression characteristics under *B. amyloliquefaciens* stress, the total RNA from *S. sclerotiorum* samples treated with the fermentation broth of *B. amyloliquefaciens* (TS) and control samples (CS) were extracted for transcriptome sequencing. Based on the comparative analysis of the Fragments Per Kilobase of transcript per Million (FPKM) mapped reads distribution and FPKM density distribution (Fig. 2), there were differences in the dispersion and population distribution of gene expression between TS and CS samples, indicating that the expression of some *S. sclerotiorum* genes were changed in response to *B. amyloliquefaciens* stress.

**Figure 3:**
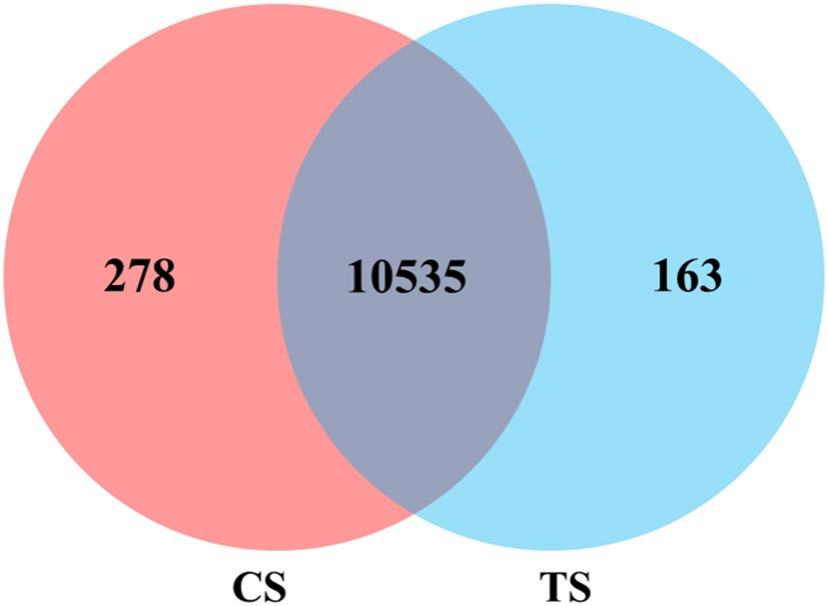
Venn diagram presenting the overlap of expressed genes between CS and TS. Pink represents CS and blue represents TS.

**Figure 4:**
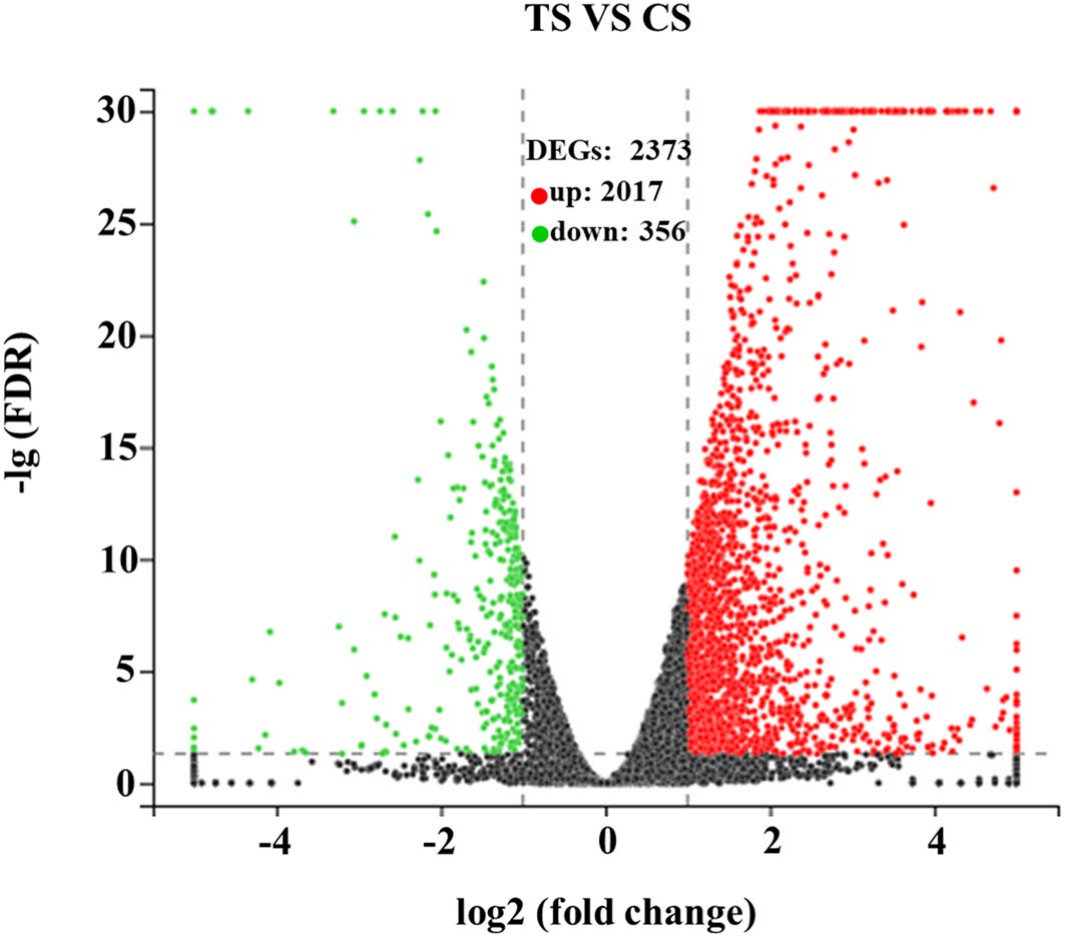
Volcano plot of DEGs between TS and CS. Splashes represent different genes, where black indicates the mean number of genes without significant differential expression, red indicates the mean of significantly upregulated genes, and green indicates the mean of significantly downregulated genes.

In total, 10,813 expressed genes were present in CS, and 10,698 were present in TS. Among them, 10,535 genes were co-expressed across all samples, of which 278 and 163 were specifically expressed in CS and TS, respectively (Fig. 3). Expressed genes with a fold change ≥ 2 and a false discovery rate (FDR) < 0.05 were designated as differentially expressed genes (DEGs). Overall, 2,373 DEGs were identified in TS vs. CS. Among them, 2,017 genes were upregulated and 356 genes were downregulated (Fig. 4).

### Experimental validation of gene expression using qRT-PCR

In order to validate the reliability of the DEGs data obtained from transcriptome analysis, the expression level of 20 genes, including 15 upregulated and 5 downregulated genes, were selected for qRT-PCR analysis. Each assay was performed in triplicate with *Actin* and *Tubulin* as the internal reference genes. The expression patterns of 20 genes analyzed by qRT-PCR were consistent with the RNA-Seq analysis (Table 1). qRT-PCR analysis results therefore confirmed that the transcriptome analysis was reliable.

**Table 1:** qRT-PCR primers used in this study to validate the RNA-Seq data.

Genes	Primers (5′–3′)	Log2FC (RNA-Seq)	Log2FC (*Actin*)	Log2FC (*Tubulin*)
*sscle_16g109520*	F: TGCCGTTTACTCTGGTGGTC R: AGATGGGTTGGTGGTTCCTTC	1.55573	1.36216	1.65070
*sscle_02g019140*	F: GTATGCGGAGCGGAGATTGT R: TGGAGGGGATTGAGTTGTGG	1.72783	1.40369	1.69224
*sscle_04g034440*	F: CCACGGAGAGAATGGATGAGA R: GGACTAACAGGACTAACGGGAC	2.18592	1.64946	1.93800
*sscle_06g049790*	F: CTAACTTCTACTCTGCCCGCT R: GTGTGTTGTGTTTGCCCAGT	2.45371	1.83677	2.12532
*sscle_10g075870*	F: GAAGTCCCTGTTCCTCCCATC R: CACCAAACGCACTACTACCAA	1.70292	0.46362	0.75217
*sscle_07g055380*	F: CCTGAGTTATGGGTCGGAGAG R: ACCACAGATTGCTTCTTGCC	1.55046	1.37108	1.65963
*sscle_02g013950*	F: GGACAAAAGGGAACTGCCGA R: ACGGGGGTTCCATCTTTGTAG	1.11719	1.08732	1.37586
*sscle_03g026200*	F: GTTGCTTTCTCGCCATCTCA R: GACGGTGGGTATCTGGGTATG	1.13554	0.92791	1.21645
*sscle_07g058850*	F: ATGGCTTTGTTTTCGGGTGG R: GGTTCCTGATGGTCTCGTGG	1.58180	1.36277	1.65132
*sscle_16g107740*	F: TCAACCTATCGAAGTATGGACG R: AATGAAGGGTGGTTTCTTGACG	3.06223	3.06322	3.35176
*sscle_05g042140*	F: GTAGAGCACCAGCAATCAAGG R: AACCAACCGCACTGTCCAA	4.72052	4.71977	5.00831
*sscle_04g033170*	F: TCCACCAAAGGCAAAGAAACC R: AAGCACCCATTACTCCCCAC	2.06681	1.45733	1.74588
*sscle_08g067600*	F: GCATTTACAGCATTGGGCGT R: TTGCCAGAGCCCGTAGGT	1.20933	1.12149	1.41004
*sscle_07g058680*	F: GCCGACAATACAAATACCACCA R: TCGTAGACCCCATCCACCTT	1.23534	1.00904	1.29759
*sscle_12g087380*	F: CAGATGAGATGGCTGCGAGT R: CTGGTCGGGTGTTTCCTTGA	1.43927	0.96316	1.25171
*sscle_16g110440*	F: CCGACGACTTAGGGGACAAC R: CAACATTCGCACGCCAGTAA	− 5.32034	− 6.31581	− 6.02726
*sscle_03g023410*	F: GCAACAATAGGATGCGGACT R: GAAACGGTGGCTTGAGAGGA	− 4.76991	− 5.08976	− 4.80121
*sscle_06g048630*	F: CCGTCAAGCGTCAACAAACC R: ACCCAAAATGGCGTAGGAGA	− 2.58260	− 2.89794	− 2.60940
*sscle_03g030810*	F: CCTTTTGCGAGTTGGTTGGT R: GCGATGTTTTTACCGAGAGCC	− 2.27651	− 2.43927	− 2.15072
*sscle_04g036160*	F: ATGCCAAAGTGGAGCGGA R: TTGAGGAGGGGCGGATAAGA	− 1.45465	− 1.68747	− 1.39892

### GO and KEGG enrichment analyses

**Figure 5:**
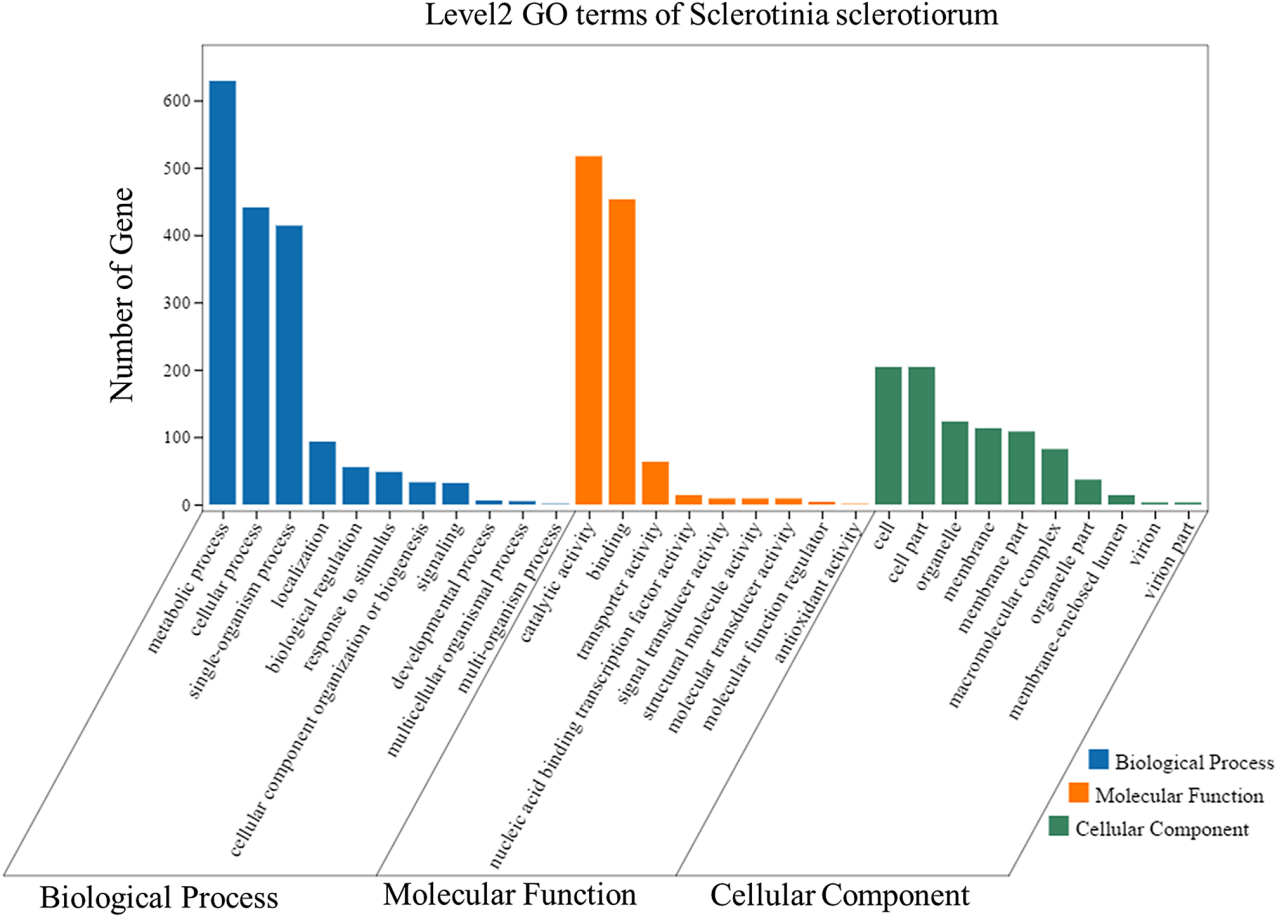
GO classifications of DEGs. The x-axis presents three major functional categories of GO terms, including biological process, cellular component, and molecular function. The y-axis presents the number of DEGs.

Gene Ontology (GO) was used to classify the functions of DEGs. A total of 2,373 DEGs were significantly categorized into 44 functional groups belonging to three GO terms, including biological process, molecular function, and cellular component. Metabolic process, cellular process, and single-organism process were the most highly represented terms among biological process. Under molecular function, category activity and binding represented the majority of DEGs. Within cellular component, cell, cells parts organelle, membrane, and membrane part were the dominant terms (Fig. 5).

**Figure 6:**
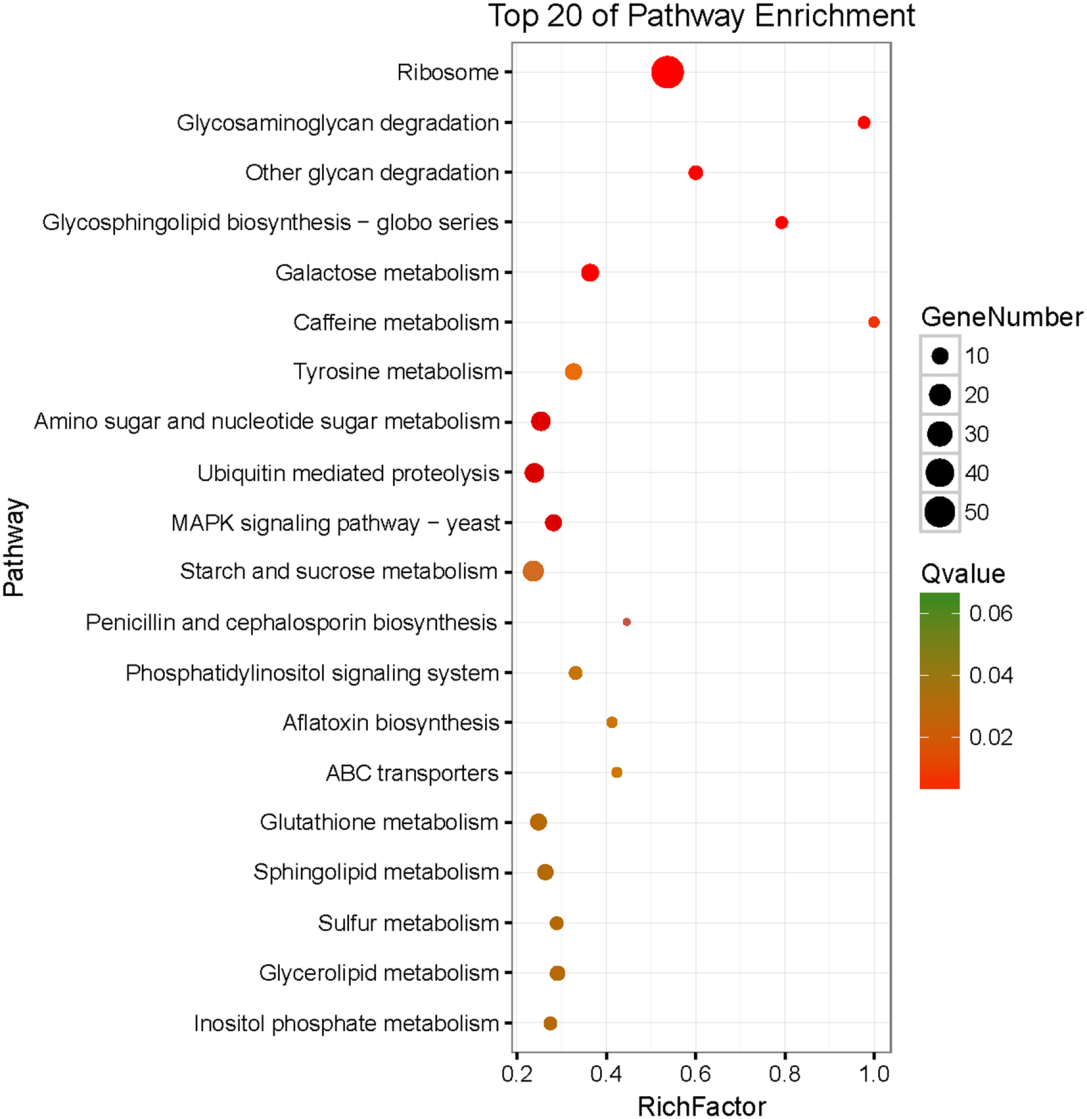
Scatter plot of enriched KEGG pathways for DEGs. The x-axis presents the percentage of DEGs belonging to the corresponding pathway. The y-axis represents the top 20 pathways. A Q value is the corrected p value.

In order to perform functional classifications and pathway assignments of *S. sclerotiorum* genes under *B. amyloliquefaciens* stress, all DEGs were analyzed against the Kyoto Encyclopedia of Genes and Genomes (KEGG) database. In total, 2,373 DEGs were successfully annotated and assigned to 106 pathways. Among them, a total of 42 pathways were significantly enriched and categorized in four branches, including genetic information processing, metabolism, environmental information processing, and cellular processes. Of which, the DEGs enriched in metabolism were dominant. The top 20 pathways with the most abundant DEGs are presented in Fig. 6. Results revealed that the number of genes involved in ribosome, starch, and sucrose metabolism, ubiquitin-mediated proteolysis, glycosaminoglycan degradation, other glycan degradation, glycosphingolipid biosynthesis-globo series, sphingolipid metabolism, and ABC transporters signalling pathway were the most abundant.

### Comparative analysis of DEGs

1. Cell wall synthesis-related genes in *S. sclerotiorum*
  Cyclic lipopeptide produced by *Bacillus* inhibited the synthesis of cell walls, resulting in weakened cell wall strength and cells that turned into "balloons" under the osmotic pressure of cell inclusions^14^. In this study, TS were also turned into "balloons", indicating that the cell walls of *S. sclerotiorum* were damaged, and may due to the stress of cyclic lipopeptides secreted by *B. amyloliquefaciens*. Cell wall synthesis-related genes were upregulated in TS (i.e., genes related to the synthesis of (1,3)-beta-D-glucan in *S. sclerotiorum*, *sscle_07g055380*, *sscle_16g109520*, and *sscle_16g109250*), suggesting that *S. sclerotiorum* increased the stability of glucan or cell walls by upregulating genes in the glucan synthesis pathway to cope with the stress of cyclic lipopeptide secreted by *B. amyloliquefaciens* (Fig. 7).

**Figure 7:**
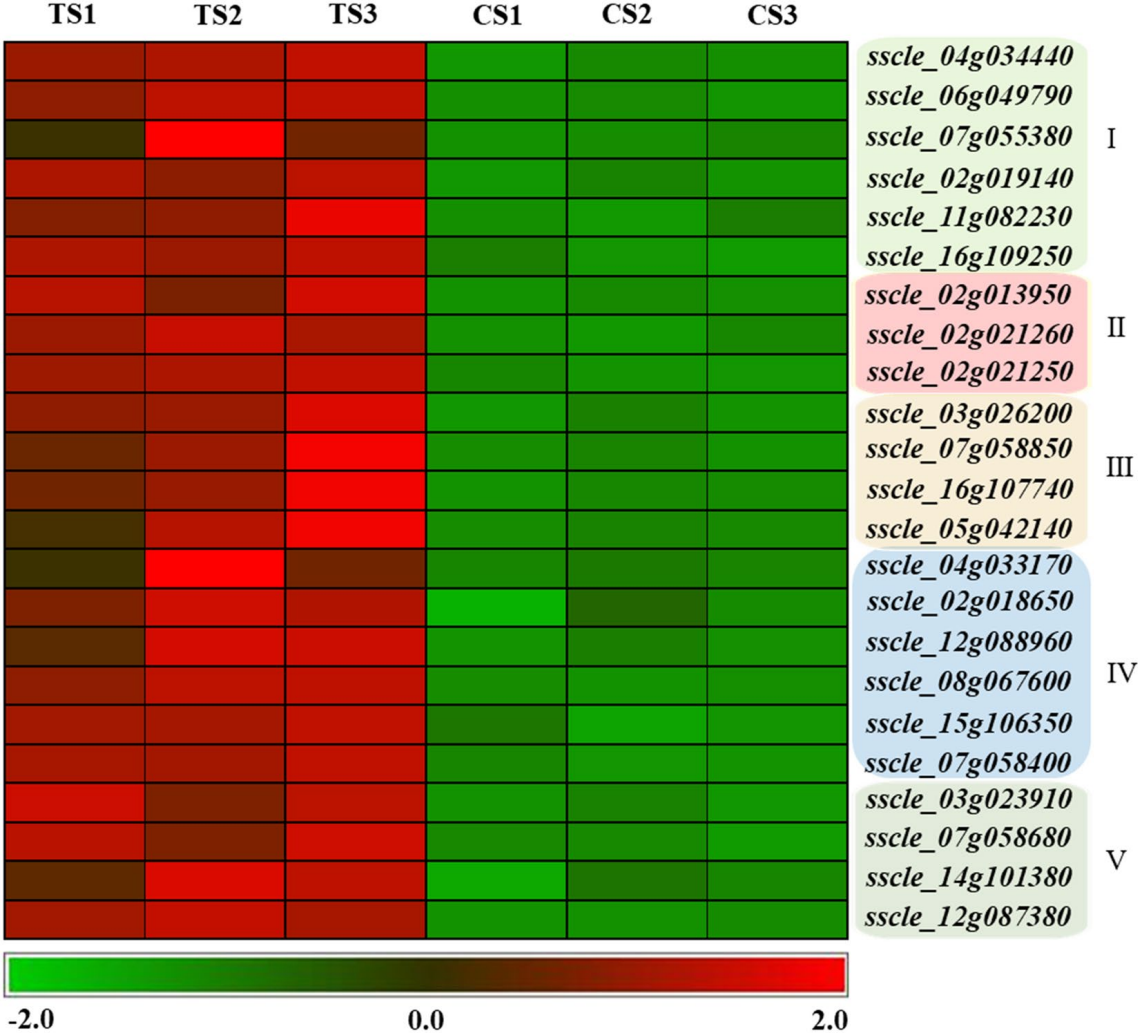
Comparative analysis of DEGs in *S. sclerotiorum*. (I) Cell wall synthesis-related genes. (II) Cell membrane synthesis and stability-related genes. (III) Antioxidant-related genes. (IV) ABC transporter-related genes. (V) Autophagy-related genes*.*
2. Cell membrane synthesis and stability-related genes in *S. sclerotiorum*
  Ergosterol is an important component of fungal cell membranes and ergosterol synthesis pathway-related enzymes are the targets of many antifungal drugs. Therefore, ergosterol synthesis pathway was also the focus of this study. In TS, the genes, *sscle_11g086600* (similar to ergosterol biosynthesis protein, ERG28, in *Botrytis cinerea* T4) and *sscle_02g013950* (similar to CYP51, eburicol 14 alpha-demethylase in *B. cinerea* T4), were significantly upregulated (Fig. 7). These genes encode key enzymes during the ergosterol synthesis, and ergosterol maintains the stability and fluidity of the cell membrane. Thus, we speculate that *S. sclerotiorum* responds to the stress of *B. amyloliquefaciens* through adjusting the stability and fluidity of cell membranes by upregulating genes in the ergosterol synthesis pathway.
  Fatty acids combined with phospholipids form an important component of the plasma membrane and maintain the fluidity of the cell membrane. Two genes (i.e., *sscle_02g021250* and *sscle_02g021260*) related to fatty acid synthesis in TS were upregulated under *B. amyloliquefaciens* stress (Fig. 7). A beta subunit of fatty acid synthetase, *sscle_02g021250*, has high homology with fatty acid synthetase (FAS1) and *sscle_02g021260* was annotated as an alpha subunit of fatty acid synthetase and has high homology with the fatty acid synthetase gene, *FAS2*. *FAS1* and *FAS2* are fatty acid synthase-related genes in yeast, which are involved in the membrane biosynthesis ^15^. Upregulation of these two genes in TS may be synergistic and alleviate the damage of antibiotic substances of *B. amyloliquefaciens* (i.e., cyclic lipopeptides) to the cell membrane.
3. Antioxidant-related genes in *S. sclerotiorum*
  Amphotericin B, an antifungal drug, causes oxidative damage to fungal cells and can lead to the death of cells^16^. Cyclic lipopeptides from *Bacillus* acts in a similar way as amphotericin B, thus, the antioxidant enzymes in *S. sclerotiorum* were a major focus of this study. The gene, *sscle_03g026200*, encodes peroxisomal catalase and the gene, *sscle_07g058850*, has high similarity with heme-binding peroxidase; both genes were significantly upregulated in TS (Fig. 7). The upregulated expression of these two genes may be a way in which *S. sclerotiorum* eliminates free oxygen stress.
  Additionally, glutathione binding and ABC transporter proteins were involved in antioxidant stress. In TS, the genes, *sscle_16g107740* and *sscle_05g042140*, which encode glutathione S-transferase, were significantly upregulated (Fig. 7). The significantly upregulated ABC transporter-related genes in TS included *sscle_12g088960*, *sscle_02g018650*, *sscle_04g033170*, *sscle_06g050350*, *sscle_07g058400*, *sscle_15g106360*, *sscle_15g106350*, *sscle_14g097690*, and *sscle_08g067600* (Fig. 7). ABC transporter proteins act as an efflux pump that remove toxic substances from the cell; these enzymes have antioxidant and detoxifying effects. By upregulating the expression of these genes, *S. sclerotiorum* eliminates reactive oxygen species (ROS) and conducts detoxification. Moreover, ABC transporter proteins can release cyclic lipopeptides outside the cell, further reducing the damage of antimicrobial substances to *S. sclerotiorum* mycelia cells due to oxygen stress.
4. Autophagy-related genes in *S. sclerotiorum*
  Autophagy pathway removes damaged organelles, such as mitochondria, and acts as an intracellular "scavengers" to maintain cellular homeostasis. A total of three upregulated genes were related to autophagy in *S. sclerotiorum*, namely *sscle_07g058680*, *sscle_14g101380*, and *sscle_12g087380*. *sscle_07g058680*, *sscle_14g101380*, and *sscle_12g087380* encode the autophagy-related proteins, ATG15, ATG22, and ATG1, respectively. ATG15 is a vacuolar lipase that functions in the breakdown of autophagic bodies, ATG22 is an amino acid permease on the vacuolar membrane, and ATG1 is a serine/threonine protein that regulates the magnitude of autophagy. The upregulated expression of these three autophagy-related genes in *S. sclerotiorum* treated with fermentation broth removed ROS, damaged cell structures and organelles, thereby ensuring the normal growth of cells (Fig. 7).

### Response pattern of *S. sclerotiorum* to *B. amyloliquefaciens* stress

**Figure 8:**
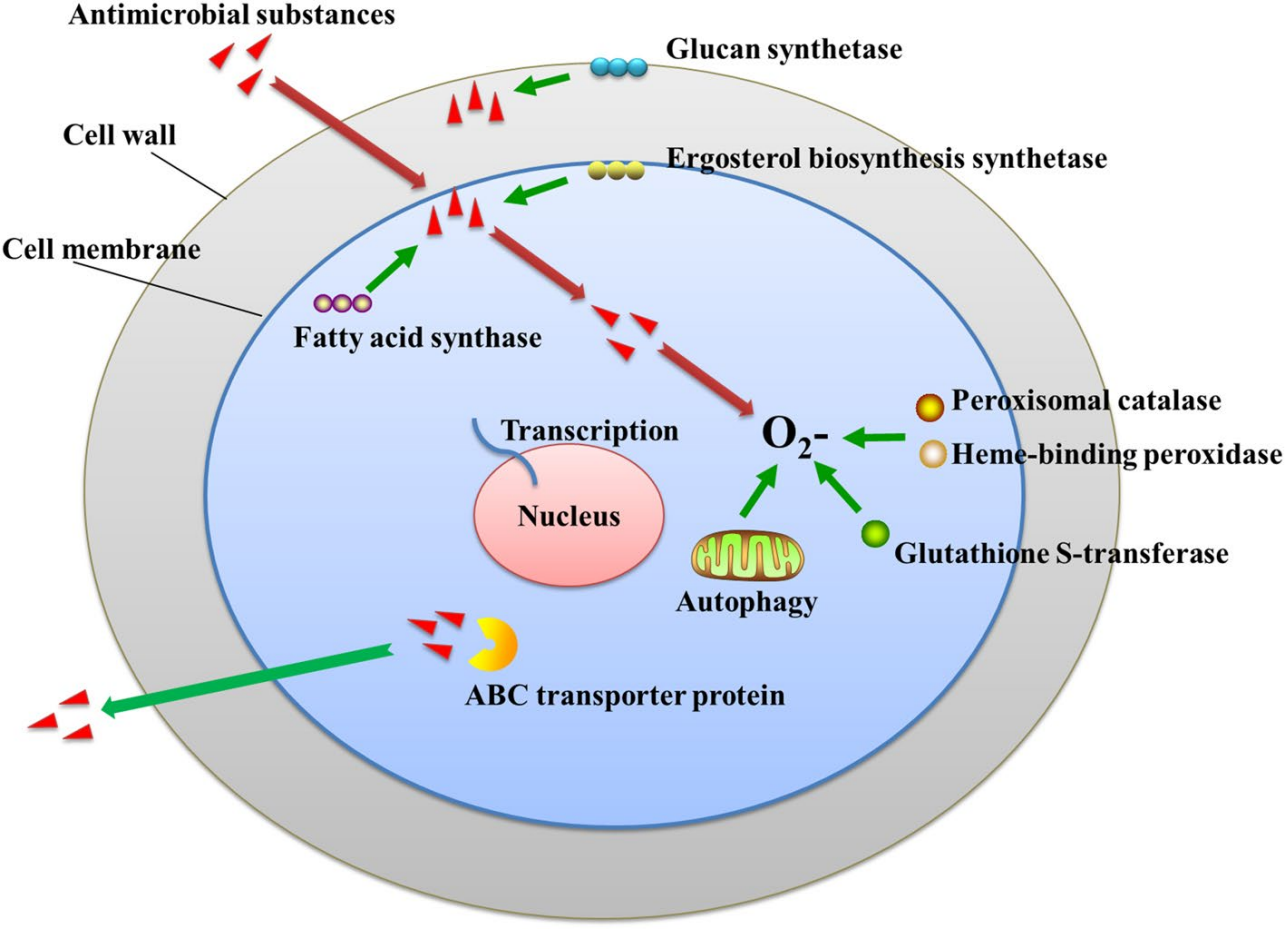
Response pattern of *S. sclerotiorum* to *B. amyloliquefaciens* stress.

The effects of *B. amyloliquefaciens* stress on *S. sclerotiorum* include the inhibition of cell wall and cell membrane synthesis, damage to the stability of the cell membrane, induction of the production of ROS, and oxidative damage to cells, among others. In order to respond to the stress of cyclic lipopeptide antimicrobial substances, *S. sclerotiorum* uses a variety of response mechanisms. In this study, the expression status of various types of *S. sclerotiorum* genes was synthesized to draw a response pattern map (Fig. 8). *S. sclerotiorum* upregulated the expression of cell wall component proteins (i.e., (1,3)-beta-D-glucan synthase) and ergosterol synthetic enzymes (i.e., ERG28 and CYP51) in order to maintain normal synthesis of the cell wall and cell membrane. *S. sclerotiorum* upregulated the expression of fatty acid synthase in response to membrane damage caused by *B. amyloliquefaciens* antimicrobial substances, thereby increasing the fluidity of the membrane and maintaining normal physiological activity of the cell. The introduction of *B. amyloliquefaciens* antimicrobial substances into cells induced the production of ROS, causing cellular damage. Therefore, *S. sclerotiorum* significantly expressed peroxisomal catalase, heme-binding peroxidase, and glutathione S-transferase in order to eliminate ROS and reduce damage. Meanwhile, increased autophagy removed ROS and damaged cell structures and organelles to ensure normal cellular growth. Moreover, the upregulation of ABC transporter proteins alleviated damage caused by antimicrobial substances and transported them outside the cells. The response pattern map revealed that *S. sclerotiorum* regulated many signalling pathways in response to *B. amyloliquefaciens* stress.

## Discussion

In this study, transcriptome sequencing was used to analyze changes in the gene expression characteristics of *S. sclerotiorum* under *B. amyloliquefaciens* stress. Results revealed that *S. sclerotiorum* alleviated damages by changing gene expression at the action site of antimicrobial substances produced by *B. amyloliquefaciens*.

Antimicrobial substances from *Bacillus* include cyclic lipopeptides and other lipopeptides, and their anti-fungal mechanisms include the inhibition of the cell wall^17^ and cell membrane synthesis^18^, and oxidative damage to the cell membrane^16,19^. Cyclic lipopeptides from *Bacillus* inhibit the activity of (1,3)-beta-D-glucan synthase in fungi, block cell wall biosynthesis, and cause the rupture of cell walls^17^. An *Aspergillus niger*-resistant strain upregulates the expression of a (1,3)-beta-D-glucan synthase gene and expresses a large number of glucan synthases to alleviate the toxic effects of caspofungin^20^. Likewise, *S. sclerotiorum* upregulates genes (i.e., *sscle_07g055380*, *sscle_16g109520*, and *sscle_16g109250*) related to the synthesis of (1,3)-beta-D-glucan to cope with the stress of cyclic lipopeptides secreted by *B. amyloliquefaciens*. However, the upregulated expression of these genes did not change the inhibition state of cell wall synthesis in *S. sclerotiorum*, and highly malformed hyphae were observed in this study. This symptom was similar to the morphological changes observed in *A. fumigatus* under the stress of pneumocandins^14^.

Ergosterol is the major component of the fungal cell membrane, which maintains membrane fluidity and permeability^15,21^. Cyclic lipopeptides from *Bacillus* possess biological activities and amphiphilic properties, which are anchored in the lipid layer of the cell membrane and destroy the integrity of biofilm^22^. Fungi upregulate the expression of genes related to the ergosterol biosynthesis in order to alleviate damages caused by ergosterol-inhibiting antifungal drugs to the cell membrane. The ergosterol biosynthesis pathway-related gene, *ERG11*, in *A. fumigatus* was upregulated under amphotericin B stress^23^. *Candida albicans* resists the inhibition of azole drugs by upregulating the ergosterol synthesis pathway gene, *ERG16*, candida drug resistance gene, *CDR1*, and multidrug resistance gene, *MDR1*^24^. Upregulation of the ergosterol biosynthesis pathway-related genes, *ERG28* and *CYP51*, may be attributed to the efforts of *S. sclerotiorum* for maintaining the biosynthesis and fluidity of the cell membrane to alleviate the toxic effects of antimicrobial substances produced by *B. amyloliquefaciens*. These results were consistent with previous findings.

Cyclic lipopeptide antibiotics from *Bacillus* bind to the lipid layer of the cell membrane, damage the structure, and induce permeability of the fungal cell membrane^25,26^. WH1fungin, a lipidpeptide produced by *B. amyloliquefaciens*, induces the formation of holes in the cell membrane of fungi at high concentrations, leads to the leakage of cell contents, and results in fungal death^27^. The leakage of cell contents in the mycelium of *S. sclerotiorum* was also observed in this study. The formation of holes in the cell membrane of *S. sclerotiorum* may be due to high concentrations of *B. amyloliquefaciens* fermentation broth and the large number of cyclic lipidpeptide antimicrobial substances. High concentrations of lipopeptides lead to complete destruction of the lipid bilayer and formation of micelles in the cell membrane, as well as a loss of fluidity of the cell membrane^9^. Some fungi maintain fluidity of the cell membrane by upregulating the expression of some genes involved in the fatty acid synthesis pathway to minimize damages to the cell membrane. Under stress of the antagonistic microbe, *Collimonas fungivorans*, *A. niger* upregulated the expression of the D9-fatty acid desaturase gene to produce unsaturated fatty acids, improve fluidity of the cell membrane, and reduce damages caused by the antimicrobial substances from *C. fungivorans*^28^. Upregulated genes (i.e., *sscle_02g021250* and *sscle_02g021260*) in TS were homologous with fatty acid synthase and participated in the biosynthesis of the cell membrane. Upregulated expression of these two genes by *S. sclerotiorum* alleviated the damage caused by cyclic lipopeptide antibiotics produced by *Bacillus* to the cell membrane and maintained its fluidity and integrity, which is similar to the antimicrobial mechanism observed in *A. niger*. These results indicated that *S. sclerotiorum* responds to *B. amyloliquefaciens* stress by regulating the expression of fatty acid synthesis pathway-related genes.

Amphotericin B induces reactive oxygen stress in fungal cells, thereby inhibiting the growth of mycelium^16,29,30^. Under caspofungin and fenpropimorph stress, *A. niger* upregulated the expression of antioxidant stress-related genes, including glutaredoxin, ABC transporter proteins, and glutathione S-transferase genes, thereby protecting cells from damage caused by ROS^20^. In this study, the expression levels of antioxidant-related genes in TS were upregulated, including the peroxisomal catalase gene, *sscle_03g026200*, and heme-binding peroxidase gene, *sscle_07g058850*, glutathione S-transferase genes, *sscle_16g107740* and *sscle_05g042140*, and ABC transporter-related genes, *sscle_12g088960*, *sscle_02g018650*, and *sscle_04g033170*. These findings were consistent with the results of Sokol-Anderson et al*.*^30^.

Peroxisomal catalase and heme-binding peroxidase are important enzymes involved in the biological antioxidant defense system, which play important roles in eliminating ROS damage^31,32^. Glutathione S-transferase is a major detoxification enzyme that plays an important role in eukaryotic drug resistance^33^. ABC transporter proteins play important roles in fungal resistance to drugs, stress responses, and cellular detoxification^34,35^. ABC transporter proteins in fungi function as membrane drainage pumps that expel drugs out of the cell and improve drug resistance^1^. Upregulation of the ABC transporter protein gene, *Pmd1p*, significantly increased resistance to leptomycin B and other fungal drugs in *Schizosaccharomyces pombe*, while *Pmd1p* deletion mutants were sensitive to drugs^36^. The excessive accumulation of ROS disrupts cellular homeostasis, resulting in oxidative stress and mitochondrial dysfunction. Autophagy reduces oxidative damage by engulfing and degrading oxidized substances^37^. Upregulation of antioxidants and autophagy-related genes in *S. sclerotiorum* alleviated oxygen stress injury and other toxic effects caused by antimicrobial substances produced by *B. amyloliquefaciens*, indicating that *B. amyloliquefaciens* causes peroxidation damage to the cell membrane.

Microorganisms are subjected to various stressors in nature and respond by altering the expression of genes in order to mitigate damage. Antifungal drugs are also a form of a stress to fungi. The different types of damage caused by antifungal drugs vary and the mechanisms of fungal drug resistance are complex. Multidrug resistance in *S. cerevisiae* is controlled by a complex multigene network regulated by the Pdr1p and Pdr3p transcription factors, which activate multiple proteins, including ABC transporters, major cotransporter superfamily permeability enzymes, and lipid metabolic pathway proteins that are directly involved in the regulation of drug resistance^38^, thereby achieving drug resistance by passivating drugs or replacing drug targets.

*S. sclerotiorum* regulated the expression of multiple genes in multiple pathways in response to cyclic lipidpeptide antimicrobial substances produced by *B. amyloliquefaciens* fermentation broth in order to alleviate or reduce the toxic effects of these substances. This model was similar to the multidrug resistance observed in *S. cerevisiae* and may be a common mechanism in fungi. Currently, drug-resistant plant pathogen strains have appeared in agricultural production, such as *B. cinerea*^39^ and *Pseudoperonospora cubensis*^40^. Although the reports on the resistance of plant pathogenic fungi to biocontrol agents were limited, under the continuous stress of biocontrol antimicrobial substances, plant pathogenic fungi may develop resistance to biocontrol agents by mitigating biocontrol agents and the damage they cause. Resistance of lepidoptera to *B. thuringiensis* suggests that the development of plant pathogenic fungi resistant to biocontrol agents should be a focus of future research^41^.

In this study, the response mode of *S. Sclerotiorum* to *B. amyloliquefaciens* stress was uncovered at the transcriptome level. These findings will serve as a foundation for further verification of the function of resistance-related genes.

## Materials and methods

### Strains and culture conditions

*Bacillus* strain Bam22 was isolated from the rhizosphere of oilseed rape in Sichuan province, China. It was identified as *B. amyloliquefaciens* by PCR amplification and sequence analysis of 16S rDNA, *gyrA and cheA* gene sequences, and grown in Luria Broth (LB) medium at 33 °C for 24 h at 170 rpm. *S. sclerotiorum* strain 1980 was cultured on PDA at 20 °C and stored in PDA slants at − 80 °C.

### Inhibitory effects of *B. amyloliquefaciens* on *S. sclerotiorum* mycelial growth

To test the inhibitory ability of *B. amyloliquefaciens* on *S. sclerotiorum* mycelial growth, *S. sclerotiorum* strain 1980 was dual cultured with *B. amyloliquefaciens* strain Bam22. A mycelial agar plug (5 mm diameter) containing actively growing 1980 hyphae was transferred from a 1-day-old PDA culture to the centre of a plate (9 cm diameter) containing 20 mL PDA. Then, 2 μL Bam22 culture was placed at a distance of 3 cm from the *S. sclerotiorum* colony. Plates were incubated at 20 °C for 5 d. Each treatment was repeated three times.

To assess the inhibition effect on fungal growth, Bam22 was cultured in LB medium at 33 °C for 36 h at 170 rpm. Bam22 fermentation broth was filtered by a 0.22 μm filter and added to PDA medium with final concentrations of 5% and 10%. LB medium was used as the control. The growth rate of *S. sclerotiorum* was measured after incubation at 20 °C for 2 d.

### Sample preparation for transcriptome sequencing

To obtain fungal mycelia, *S. sclerotiorum* was placed on cellophane membrane overlaying PDA in 90 mm diameter dishes. After incubation at 20 °C for 1 d, the cellophane membrane containing *S. sclerotiorum* strain 1980 hyphae was transferred to a PDA plate containing 10% Bam22 fermentation broth and incubated at 20 °C for 1 d. Mycelia were collected and immediately freezed using liquid nitrogen for RNA extraction with three biological replicates for each sample.

### RNA extraction and transcriptome sequencing

Total RNA isolation was conducted following the methods described by Yang et al*.*^42^. The mRNAs were enriched using Oligo(dT) beads and fragmented into shorter fragments using a fragmentation buffer and reverse transcribed into cDNA with random primers. Second-strand cDNAs were synthesized by DNA polymerase I, RNase H, dNTP, and buffer. Then, cDNA fragments were purified with a QiaQuick PCR extraction kit (Qiagen,Germany), end repaired, poly(A) added, and ligated to Illumina sequencing adapters. The ligation products were size selected by agarose gel electrophoresis, PCR amplified, and sequenced using Illumina HiSeq™ 2,500 by Gene Denove Biotechnology Co. (Guangzhou, China)^43^.

### Analysis of differentially expressed genes

In order to obtain high-quality clean reads, raw reads were further filtered by removing adapters, reads contained more than 10% unknown bases (‘N’), and low-quality reads^44^. The clean reads were mapped onto the genome *S. sclerotiorum*^45^ using Tophat2 (version 2.0.3.2) with a Bowtie2 index^46,47^ . Mismatches of no more than two bases were allowed in the alignment. The gene-expression level was calculated by software RSEM^48^ and normalized using the FPKM (fragments per kb of transcript per million mapped reads) method^49^. The edgeR package (https://www.bioconductor.org/packages/release/bioc/html/edgeR.html) was used to identify the differentially expressed genes (DEGs) between wild-type strain 1980 and strain 1980 treated with the fermentation broth of *B. amyloliquefaciens*. We identified genes with a fold change ≥ 2 and a false discovery rate (FDR) < 0.05 in a comparison as significant DEGs. The DEGs were then subjected to enrichment analysis of Gene Ontology (GO) and KEGG pathways.

### GO and KEGG pathway enrichment analyses of DEGs

GO and KEGG pathway enrichment analysis was performed using the OmicShare tools, a free online platform for data analysis (www.omicshare.com/tools). Significantly enriched GO terms and KEGG pathway in DEGs comparing to the *S. sclerotiorum* genome background were defined by hypergeometric test with FDR ≤ 0.05 as a threshold.

### qRT-PCR validation

In order to validate the reliability of DEGs data obtained from the transcriptome sequencing of *S. sclerotiorum*, qRT-PCR was conducted on 20 DEGs with *Actin* and *Tubulin* as the internal reference genes. The same batch of sequenced RNA samples was used, and the first-strand cDNA was synthesized using a Super RT kit (TaKaRa, Dalian, China) following the manufacturer’s instructions. qRT-PCR was conducted using a CFX96 Real-Time PCR Detection System (Bio-Rad, CA, USA) with iTaq universal SYBR Green supermix (Bio-Rad, CA, USA). Relative expression was calculated using the 2^-△△Ct^ method with three independent replicates^42^ .

## Data Availability

All raw sequence data are stored in the Sequence Read Archive (SRA) database (Accession Nos.: SRR10297529–SRR10297534).
