# Author Correction: Comparative transcriptome analysis of *Sclerotinia sclerotiorum* revealed its response mechanisms to the biological control agent, *Bacillus amyloliquefaciens*

**DOI:** 10.1038/s41598-021-84382-8

**Published:** 2021-02-25

**Authors:** Xiaoxiang Yang, Lei Zhang, Yunjia Xiang, Lei Du, Xiaoqin Huang, Yong Liu

**Affiliations:** 1 Institute of Plant Protection, Sichuan Academy of Agricultural Sciences, Chengdu, 610066 Sichuan People’s Republic of China; 2 Key Laboratory of Integrated Pest Management On Crops in Southwest, Ministry of Agriculture and Rural Affairs, Chengdu, 610066 Sichuan People’s Republic of China

Correction to: *Scientifc Reports* 10.1038/s41598-020-69434-9, published online 28 July 2020

The original version of this Article contained an error.

The heading for Figure 5 was incorrect. The text,

“Level2 GO terms of Melopsittacus_undulates”.

now reads:

“Level2 GO terms of Sclerotinia sclrotiorum”.

This has now been corrected in the PDF and HTML versions of the Article.

